# Efficacy of Pulsatilla saponin B4 for treatment dairy cows affected with clinical mastitis

**DOI:** 10.1371/journal.pone.0331151

**Published:** 2025-09-02

**Authors:** Hao Zhang, Dan Shao, Zhen Yang, Shengyi Wang, Yue Zhang, Haoyu Peng, Zhetong Su, Yong Zhang

**Affiliations:** 1 College of Veterinary Medicine, Gansu Agricultural University, Lanzhou, China; 2 Key Laboratory of Veterinary Pharmaceutical Development of Ministry of Agriculture, Key Laboratory of New Animal Drug Project, Lanzhou Institute of Husbandry and Pharmaceutical Sciences, Chinese Academy of Agricultural Sciences, Lanzhou, China; 3 Chengdu Xin Hai Ying Lu Biological Medicine Technology Co., Ltd, Chengdu, China; University of Agriculture Faisalabad, PAKISTAN

## Abstract

In order to explore the therapeutic effect of natural compound Pulsatilla saponin B4 (PSB4) on clinical mastitis (CM) in dairy cows, 40 dairy cows with CM and 20 healthy dairy cows were selected as experimental samples in a large dairy farm. The CM cows were randomly divided into two groups (20 cows in each group), namely the PSB4 group treated with PSB4 neck intramuscular injection and the positive drug (PC) group treated with ceftiofur sodium neck intramuscular injection as a positive control. Another 20 healthy dairy cows were neck intramuscular injected with the same amount of normal saline to serve as a normal control (NC) group. The treatment duration was 7 days. The body temperature and milk yield of all cows were recorded on days 0, 4 and 7, while blood and milk samples were collected for the determination of relevant laboratory indicators, including somatic cell count (SCC), pathogenic microorganisms, immune factors, cytokines, antioxidant indexes, hematological and biochemical indexes. The results showed that PSB4 effectively restored the body temperature of diseased cattle, reduced SCC, significantly increased milk yield, effectively inhibited pathogenic bacteria, and significantly restored the levels of serum C-reactive protein (CRP), interleukin-1α (IL-1α), interleukin-1β (IL-1β), interleukin-6 (IL-6), interleukin-8 (IL-8), tumor necrosis factor-α (TNF-α); immunoglobulin A (IgA), immunoglobulin G (IgG), immunoglobulin M (IgM); total antioxidant capacity (T-AOC), superoxide dismutase (SOD), malondialdehyde (MDA), glutathione peroxidase (GSH-Px) and other indicators. In addition, some indicators of blood routine and blood biochemistry also returned to normal levels. In summary, PSB4 has a significant therapeutic effect on CM in dairy cows, and has a similar effect to the positive drug, which can provide a new strategy for reducing the use of antibiotics in dairy cattle farms.

## Introduction

Mastitis is one of the common diseases affecting the health of cattle and milk quality, seriously limiting the development of dairy farming worldwide. Usually, mastitis is usually divided into clinical mastitis and subclinical mastitis according to clinical signs [1]. CM has obvious pathological features, including breast swelling, signs of pain and redness, reduced milk production, changes in milk appearance, and increased body temperature in dairy cows [2–5]. And CM is the main reason for the decline in milk production and quality, the culling of affected cows, the increase in mortality and treatment costs related to mastitis, and the considerable economic losses to the dairy farming and dairy industry [6,7]. The etiology of CM is very complex, such as trauma, poor milking technology, poor environmental hygiene, genes [8,9], improper feeding and management, hormone disorders and breast defects, etc, usually a combination of reproductive environment and pathogenic microorganisms. The infection of pathogenic microorganisms is the main cause of CM, among which *Escherichia coli*, *Streptococcus*, *Staphylococcus*, *Mycoplasma bovis*, *Bacillus* and mixed infection are the most common [10–13]. Currently, the treatment of CM mainly relies on antibiotics [14–16]. However, the irregular and even abusive use of antibiotics has led to an increase in pathogen resistance and drug residues [17], posing a serious threat to public health security [18,19]. Under the global initiative to reduce the use of antibiotics, more and more studies are tending to use natural therapies, such as the use of natural herbs, natural compounds and probiotics to treat cow mastitis [20,21]. Due to the characteristics of natural products with low toxicity, low residue and a variety of biological activities, it has gradually become a research hotspot [22,23], and will be a new strategy for the treatment of CM.

Saponins are extracted from different parts of plants such as seeds, roots, stems, and leaves. In recent years, many studies have shown that saponins have a variety of biological activities, including immune regulation, anti-inflammatory, anti-oxidation and hypoglycemic effects. Such as Ginsenoside Rb2 has a good immunomodulatory effect to improve the immunity of mice. [24], Panax Noto ginseng Flower Saponins showed a good anti-inflammatory activity [25], Aralia taibaiensis saponins have significant antioxidant activity, Albizia adianthifolia root saponins have inhibitory effects on a variety of bacteria [26], Ginsenoside Rg3 can reduce tumor volume and significantly enhance anti-tumor activity [27], and Momordica charantia Saponins can effectively reduce blood sugar [28]. These results suggest that plant saponins have great potential in immune regulation and disease treatment.

**Fig 1:**
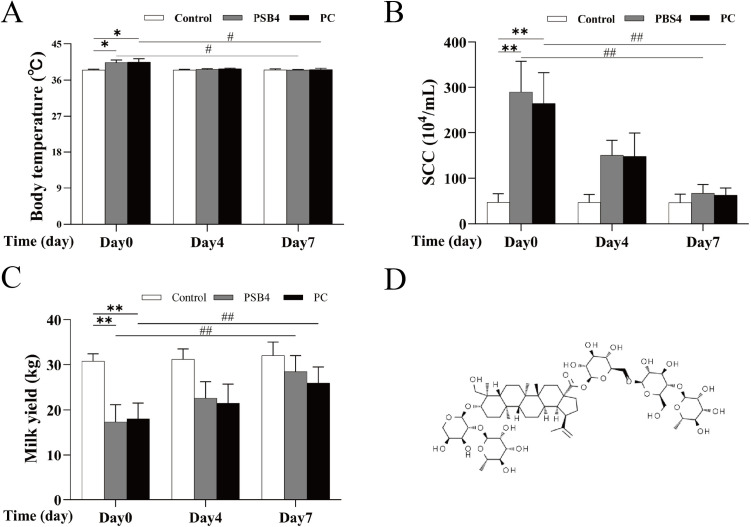
Effect of PSB4 and PC on Body temperature, SCC, and Milk yield in dairy cows. (A) Changes in body temperature. (B) Changes in somatic cell count. (C) Changes in milk production. (D) PSB4 structural formula. * *p* < 0.05, ** *p* < 0.01 vs. the NC group.^#^ *p* < 0.05, ^##^ *p* < 0.01 vs. the Before treatment (Day 0).

PSB4, a natural triterpenoid saponin compound, is isolated from the roots of *Pulsatilla chinensis,* and the formula is C_59_H_96_O_26_ (Fig 1D). Recent studies have shown that PSB4 has antibacterial, antiviral, anti-tumor, and anti-inflammatory activities [29,30]. Kang et al. research found that PSB4 can improve LPS-induced kidney and lung inflammation damage in mice by inhibiting NF-κB pathway [31]. Wang et al. research findings found that PSB4 exerts the protective effect against cisplatin-induced acute kidney injury through NF-κB pathway in mice [32]. Ma et al. study found that PSB4 can prevent acute ulcerative colitis by inhibiting TLR4/NF-κB/MAPK signaling pathway [33]. Liang et al. study found that PSB4 can alleviate colitis by reprogramming macrophage function [34]. In addition, Zhang et al. quantitative proteomic analyses discovered that 56 proteins are significantly altered by PSB4 in the 2, 4, 6-trinitrobenzene sulfonic acid-induced rats. Among them, there are proteins closely related to inflammation [35]. However, little is known about the anti-inflammatory effect of PSB4 on mastitis. Therefore, these beneficial pharmacological effects may meet the requirements for mastitis treatment in dairy cows. We used PSB4 to treat CM, evaluated its clinical signs, and compared the milk yield, SCC, pathogenic bacteria in milk samples, hematological indexes, serum biochemical indexes, antioxidant indexes, inflammatory factors and immune factors of dairy cows before and after treatment, the aim is to clarify the therapeutic effect of PSB4 on CM and provide a new strategy for the treatment of CM.

## Materials and methods

### Institutional review board statement

The study was conducted according to the guidelines of the Declaration of Helsinki and approved by the Institutional Animal Care and Use Committee of the Lanzhou Institute of Husbandry and Pharmaceutical Sciences of the Chinese Academy of Agri-cultural Sciences (Approval No. 2024-55). In addition, after the experimental party and the person in charge of the dairy farm fully communicated, they clearly knew the purpose, method and process of this animal experiment, as well as the possible effects on the cows. They also verbally agreed to carry out the animal experiments for the treatment of CM in the dairy farm and promised to fully co-operate with the experimental team to ensure that the experiments would be carried out smoothly. At the same time, the experimental party strictly followed the animal welfare related regulations to ensure that the dairy herd was properly treated.

### Study population and enrollment/exclusion criteria

The cows in this study were from a large intensive dairy farm (36 19’N, 103 18’E) in Gansu Province, China, the dairy herd has 5,000 registered cows, all cows were Holstein dairy cattle. The experimental cows were judged by the herd veterinarian according to the typical signs of CM (Includes acute, sub-acute and chronic).

#### CM determination criteria.

The diseased milk area is red and swollen, the surface temperature is high, the mammary gland is swollen, the milking is extremely sensitive, and the milk production is significantly reduced or even stopped, and the milk is abnormal, pale yellow or light red, thin or sticky flocculent, severe cases can squeeze out blood milk.

#### Enrollment criteria.

A total of 46 mastitis cows and 21 healthy cows were screened at the beginning of the trial, the selected cows had similar body weight and lactation days, aged 3–5 years old, and had a parity number ranging from 2 to 4, and mastitis cows have typical CM signs.

#### Exclusion criteria.

Dairy cows with other diseases, with antibiotics or other drugs used within 30 days, and with severe mastitis were not included in the experiment. In this experiment, 2 cows had extremely serious mastitis and the milk area was scrapped, 4 dairy cows with mastitis were treated with antibiotics or other drugs, a total of 6 cows that were all excluded from the trial. A healthy cow was excluded due to receiving antibiotic treatment. Finally, 40 CM cows and 20 healthy cows were included in the experiment.

### Study design

Register the ear tag codes of 40 CM cows that have been screened into the group, and they were divided into two groups by using the random function of SPSS software (version 24.0; IBM Corporation, Armonk, NY, USA), with 20 cows in each group: the PSB4 group and PC group. Twenty healthy cows were selected as the NC group. The PSB4 group was neck intramuscular injected with PSB4 (Guangxi Yingluweite Pharmaceutical Co., Ltd., 20240301R, 66.7%) at a dose of 0.05 mL/kg body weight, once a day. The PC group was neck intramuscular injected with ceftiofur sodium (Hebei Yuanzheng Hemu Pharmaceutical Co., Ltd., JIK240102,85%) at a dose of 1.5 mg/kg body weight per day, once a day. NC group was neck intramuscular injected with the same dosage of normal saline as PSB4 group, once a day. All cows were treated for 7 days, during the treatment, only test drugs were used, and no other drugs were added, and the blood and milk samples were collected on days 0, 4 and 7.

### Sample collection

When collecting samples, all experimental cows were fixed in a card slot. Then the rectal temperature was measured and recorded using an electronic thermometer. At the same time, milk samples and tail vein blood samples were collected (Blood samples were placed in EDTA anticoagulant tubes and non-anticoagulant tubes, respectively.), and were placed in a refrigerator (2 ~ 8°C) with an ice bag, transported to the laboratory for related index detection.

### Detection of SCC and pathogenic bacteria

The milk yield data of experimental cows were retrieved from the pasture management system. SCC in milk samples were measured using a somatic cell analyzer (Denmark, Fossomatic TM 7). Pathogenic microorganisms in milk were determined according to the instructions of the 16-unit bovine mastitis pathogen nucleic acid detection kit (Shenzhen Bioeasy Biotechnology Co., Ltd., China).

### Detection of blood routine, blood biochemical indexes, inflammatory factors, immune factors and antioxidant indexes

Blood samples in EDTA anticoagulant tubes were analyzed using an automatic blood routine analyzer (USA, VetScan HM5). Blood biochemical indices were measured using a BS-240Vet automatic clinical chemical analyzer (Shenzhen Mindray Animal Medical Technology Co., Ltd.). The blood samples in the non-anticoagulant tubes were placed at room temperature for 2 h, centrifuged at 4000 g for 5 min, and the supernatant was collected for the detection of serum related indexes. Inflammatory marker (CRP, IL-1α, IL-1β, IL-6, IL-8, and TNF-α) and immune factors (IgA, IgG, and IgM) in serum were measured using ELISA kits according to the manufacturer’s instructions (Nanjing Jiancheng Bioengineering, China). T-AOC, SOD, MDA, and GSH-Px are measured using an antioxidant index determination kit (Nanjing Jiancheng Bioengineering, China).

### Statistical analysis

SPSS software (version 24.0; IBM Corporation, Armonk, NY, USA) was used for statistical analysis. All the variables of the test indicators are in accordance with the normal distribution. No logarithmic transformation was performed on the data. The data are expressed as the mean ± standard deviation (SD). Independent sample t test was used to compare the differences between the two groups. *p* < 0.05 was considered statistically significant.

## Results

### Clinical therapeutic effect

As shown in Table 1, compared with the PC group, the PSB4 group had a higher cure rate and a lower recurrence rate. These results indicate that PSB4 is effective in the treatment of CM and is not easy to relapse.

**Table 1:** Statistic on the clinical effects of PSB4 and PC in the treatment of CM.

Group	Number of treatments	Number				Cure rate (%)	Total efficiency (%)	Recurrence rate (%)
		Cure				Effective	Invalid	Recurrence
**PSB4 group**	20	15	4	1	2	75.0	95.0	10.0
**PC group**	20	14	4	2	3	70.0	90.0	15.0

Notes: Clinical efficacy criteria: clinical recovery, the signs of redness, swelling, heat and pain in the diseased milk area disappeared completely, the milk returned to normal color by visual observation, and the milk yield basically recovered; Clinical effective, the signs of redness, swelling, heat and pain in the affected milk area are alleviated or partially disappeared, the milk color is normal or there is a small amount of floccules, and the lactation volume is increased. Clinical invalid, the signs of redness, swelling, heat and pain in the affected milk area were not significantly improved or aggravated, the milk yield continued to decline, and the milk color was abnormal. Treatment time: 7 days.

### Body temperature, SCC and milk yield

As depicted in Fig 1, 7 days after treatment, the body temperature, SCC and milk yield of cows in PSB4 group and PC group were basically restored to the level of NC group (*p* < 0.01), but the milk yield of PSB4 group was higher than that of PC group after treatment (*p* < 0.01). It is indicated that PSB4 and ceftiofur sodium have noticeable therapeutic effect on clinical mastitis, but PSB4 is superior to antibiotics in restoring milk yield of CM cows.

### Pathogenic microorganisms

Table 2 shows that clinical mastitis was mainly caused by environmental pathogenic bacteria, including *Escherichia coli*, *Enterococcus*, and yeast, followed by infectious pathogenic bacteria *Streptococcus agalactiae* and *Staphylococcus aureus*. After treatment, the negative conversion rate of pathogenic bacteria in PSB4 group and PC group was similar, and the effect was obvious. PSB4 had more significant antibacterial ability than PC on *Enterococcus* and *Escherichia coli.* In addition, PSB4 had noticeable inhibitory effect on yeast, *Streptococcus agalactiae*, and *Staphylococcus aureus*. These results indicate that PSB4 has good antibacterial properties, which is comparable to the PC drug.

**Table 2:** Effect of PSB4 and PC on the pathogenic microorganisms in milk of clinical mastitis cows.

Group & treatment	Pathogenic microorganism	Before treatment 0st day (heads)	Post treatment 7th day (heads)	Negative rate (%)
**PSB4 group**	*Corynebacterium bovis*	5	2	60.0
	*Enterococcus*	20	4	80.0
	*Escherichia coli.*	20	4	80.0
	*Saccharomyces cerevisiae*	16	3	81.2
	*Streptococcus agalactiae*	8	3	62.5
	*Staphylococcus aureus*	6	2	66.7
	*Mycoplasma bovis*	5	2	60.0
**PC group**	*Corynebacterium bovis*	4	2	50.0
	*Enterococcus*	20	6	70.0
	*Escherichia coli.*	19	5	73.7
	*Saccharomyces cerevisiae*	16	6	62.5
	*Streptococcus agalactiae*	11	3	72.7
	*Staphylococcus aureus*	7	2	71.4
	*Mycoplasma*	4	2	50.0
	*Arcanobacterium pyogenes*	2	1	50.0

Notes: Pathogenic microorganisms in milk were determined using a 16-unit bovine mastitis pathogen nucleic acid detection kit. Negative rate (%) = (The number of positive bacteria before treatment – The number of positive bacteria after treatment)/The number of positive bacteria before treatment.

### Levels of immune factors in serum

**Fig 2:**
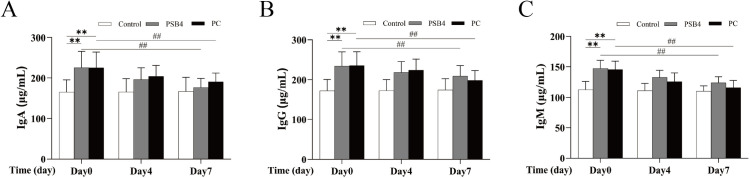
Effect of PSB4 and PC on immune factors in serum of dairy cows. (A) Changes in serum IgA levels. (B) Changes in serum IgG levels. (C) Changes in serum IgM levels. * *p* < 0.05, ** *p* < 0.01 vs. the NC group. ^#^ *p* < 0.05, ^##^ *p* < 0.01 vs. the Before treatment (Day 0).

As shown in Fig 2, the levels of immune factors (IgA, IgG, IgM) in serum of dairy cows in PSB4 and PC groups were higher than those in NC group on day 0 (*p* < 0.05, *p* < 0.01). With the increase of treatment days, the contents of IgA, IgG and IgM of PSB4 and PC groups decreased significantly, and the content of IgA in both groups returned to the level of NC group. These findings reveal that PSB4 can reduce the content of immune factors and alleviate immune imbalance, and has the same therapeutic effect as PC drug.

### Levels of cytokines in serum

**Fig 3:**
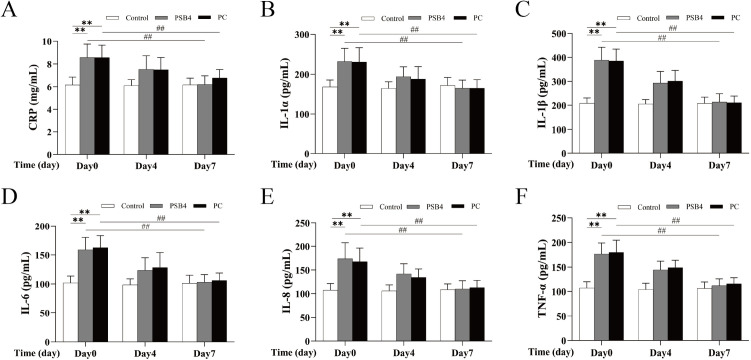
Effect of PSB4 and PC on inflammatory factors in serum of dairy cows. (A) Changes in serum CRP levels. (B) Changes in serum IL-1α levels. (C) Changes in serum IL-1β levels. (D) Changes in serum IL-6 levels. (E) Changes in serum IL-8 levels. (F) Changes in serum TNF-α levels. * *p* < 0.05, ** *p* < 0.01 vs. the NC group. ^#^ *p* < 0.05, ^##^ *p* < 0.01 vs. the Before treatment (Day 0).

As shown in Fig 3, after 7 days of treatment, the levels of inflammatory factors (CPR, IL-1α, IL-1β, IL-6, IL-8, TNF-α) in serum of dairy cows of PSB4 group and PC group were decreased. On the 7th day, except that the CPR level in PC group was higher than that in NC group (*p* > 0.05), the contents of other inflammatory factors (IL-1α, IL-1β, IL-6, IL-8, TNF-α) in PSB4 group and PC group returned to the level of NC group. The results showed that PSB4 could reduce the level of inflammatory factors in serum of CM cows and inhibit the development of inflammation, and its therapeutic effect was comparable to that of PC drug.

### Levels of antioxidant indexes in serum

**Fig 4:**
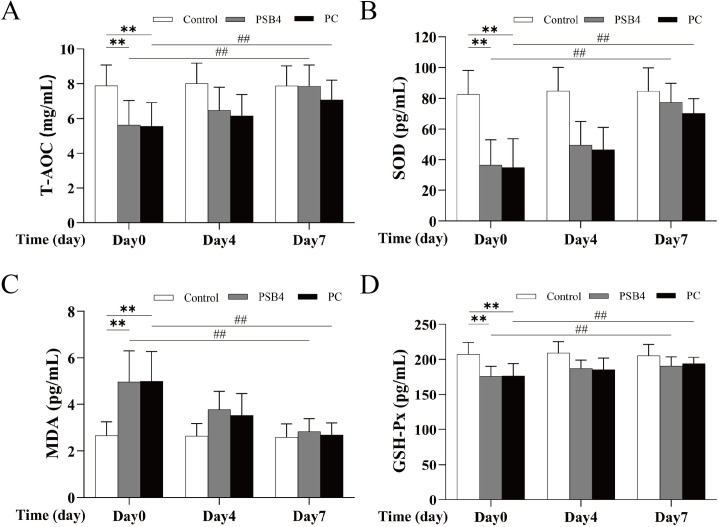
Effect of PSB4 and PC on antioxidant indexes in serum of dairy cows. (A) Changes in serum T-AOC levels. (B) Changes in serum SOD levels. (C) Changes in serum MDA levels. (D) Changes in serum GSH-Px levels. * *p* < 0.05, ** *p* < 0.01 vs. the NC group. ^#^ *p* < 0.05, ^##^ *p* < 0.01 vs. the Before treatment (Day 0).

As shown in Fig 4, after 7 days of treatment, the levels of T-AOC, SOD and MDA in serum of dairy cows in PSB4 group returned to the level of NC group, but the levels of T-AOC, SOD and GSH-Px in PC group were still different from those in NC group (*p* < 0.05, *p* < 0.01). These results showed that PSB4 improved the antioxidant capacity of CM cows, and the effect was better than that of the PC drug.

### Hematological and biochemical indexes

As shown in Table 3 and Table 4, before treatment, the contents of white blood cell (WBC), lymphocyte (LYM) and neutrophilic granulocyte (NEU) in PSB4 and PC groups were higher than those in the NC group (*p* < 0.01), while the red blood cell (RBC) content was lower than that in the NC group (*p* < 0.05). However, with the increase of treatment days, WBC, LYM and NEU these three indicators gradually returned to the level of NC group. In addition, the albumin (ALB) levels in the PSB4 and PC groups were lower than those in the NC group before administration (*p* < 0.05), and were close to those in the NC group after treatment. These results indicate that PSB4 can inhibit the development of inflammation in CM dairy cows, and has the same effect as the positive drug.

**Table 3:** Effect of PSB4 and PC on hematological parameters in dairy cows.

Index	Before treatment (0st day)			Post treatment (7st day)		
	NC	PSB4	PC	NC	PSB4	PC
**WBC (10** ^**9**^ **/L)**	7.98 ± 1.83	13.08 ± 2.22^**^	13.41 ± 2.19^**^	8.33 ± 1.43	8.53 ± 1.34	8.09 ± 1.47
**LYM (10** ^**9**^ **/L)**	4.69 ± 1.04	6.88 ± 0.94^**^	7.15 ± 0.46^**^	4.61 ± 1.08	5.01 ± 0.84	4.68 ± 0.74
**NEU (10** ^**9**^ **/L)**	2.36 ± 0.47	5.24 ± 1.44^**^	5.32 ± 1.93^**^	2.45 ± 0.66	2.49 ± 1.23	2.45 ± 1.41
**EOS (10** ^**9**^ **/L)**	0.31 ± 0.10	0.35 ± 0.19	0.29 ± 0.18	0.28 ± 0.10	0.31 ± 0.15	0.26 ± 0.13
**BAS (10** ^**9**^ **/L)**	0.23 ± 0.06	0.19 ± 0.11	0.22 ± 0.10	0.22 ± 0.11	0.24 ± 0.09	0.23 ± 0.12
**RBC (10** ^**12**^ **/L)**	7.38 ± 0.61	6.31 ± 1.17^*^	6.34 ± 0.68^*^	7.49 ± 0.43	7.03 ± 0.63	6.87 ± 0.78
**HGB (g/dL)**	11.23 ± 2.41	10.04 ± 1.68	10.78 ± 1.83	11.07 ± 2.13	10.41 ± 1.89	10.27 ± 1.83
**MCV (fL)**	54.60 ± 14.77	52.20 ± 5.87	51.10 ± 9.82	51.13 ± 12.15	50.75 ± 7.95	51.75 ± 9.51
**MCHC (g/dL)**	31.65 ± 2.54	33.48 ± 2.18	33.26 ± 2.07	33.35 ± 2.37	33.12 ± 2.37	33.9 ± 2.74
**PLT (10** ^**11**^ **/L)**	4.88 ± 2.08	4.92 ± 1.74	5.12 ± 1.91	4.88 ± 2.02	5.05 ± 1.26	5.07 ± 1.87
**MPV (fL)**	6.91 ± 0.93	6.79 ± 1.32	7.08 ± 1.10	7.17 ± 0.99	7.21 ± 0.84	7.00 ± 1.11

Notes: Data were presented as means ± SD (n = 20). ^*^p < 0.05, ^**^p < 0.01 *vs.* NC group. WBC: white blood cell; LYM: lymphocyte; NEU: neutrophilic granulocyte; EOS: eosinophil BAS: basophilic granulocyte; RBC: red blood cell; HGB: hemoglobin; MCV: mean corpuscular volume; MCHC: mean corpuscular hemoglobin concentration; PLT: platelet; MPV: mean platelet volume.

**Table 4:** Effect of PSB4 and PC on biochemical indexes in serum of dairy cows.

Index	Before treatment (0st day)			Post treatment (7st day)		
	NC	PSB4	PC	NC	PSB4	PC
**ALB (mg/mL)**	34.19 ± 8.57	23.29 ± 5.59^**^	22.95 ± 5.96^**^	33.83 ± 7.80	33.15 ± 9.22	30.29 ± 5.13^**^
**ALP (pg/mL)**	42.39 ± 20.33	38.95 ± 14.80	41.39 ± 17.07	41.62 ± 18.84	39.11 ± 13.58	41.25 ± 15.45
**ALT (pg/mL)**	29.58 ± 9.38	26.43 ± 8.42	29.00 ± 9.80	29.24 ± 9.50	26.37 ± 7.71	28.51 ± 9.57
**AST (pg/mL)**	79.46 ± 15.78	83.09 ± 10.01	84.77 ± 10.12	81.56 ± 16.27	84.17 ± 11.32	86.15 ± 12.38
**UREA (pg/mL)**	6.00 ± 1.10	6.19 ± 1.29	6.10 ± 1.14	5.96 ± 0.94	6.39 ± 1.10	6.12 ± 1.08
**CREA (pg mL)**	64.08 ± 12.01	65.60 ± 12.78	65.15 ± 13.03	64.13 ± 12.06	63.78 ± 12.08	67.24 ± 12.31
**TP (pg/mL)**	66.15 ± 11.51	71.44 ± 10.13	69.73 ± 7.69	64.13 ± 12.06	63.78 ± 12.08	67.24 ± 12.31
**LDH (pg/mL)**	81.79 ± 11.61	85.64 ± 12.04	86.11 ± 11.81	82.29 ± 10.64	85.37 ± 12.22	86.21 ± 12.50
**CK (pg/mL)**	14.55 ± 2.21	14.55 ± 2.45	14.00 ± 1.91	14.24 ± 2.07	14.11 ± 2.31	13.98 ± 2.01

Notes: Data were presented as means ± SD (n = 20). ^*^p < 0.05, ^**^p < 0.01 *vs.* NC group. ALB: albumin; ALP: alkaline phosphatase; ALT: abbreviations; AST: alanine aminotransferase; UREA: urea content in blood; CREA: creatinine; TP: total pro1tein; LDH: Lactic dehydrogenase; CK: Creatine kinase.

## Discussion

Previous studies have shown that a large number of traditional Chinese medicinal herbs can effectively inhibit pathogenic bacteria, such as *Escherichia coli*, *Bacillus*, and *Streptococcus* [36,37]; significantly reduce milk SCC [38]; reduce inflammatory response and have a good effect on the treatment of mastitis [39–41]. However, the effective plant-derived saponins for treating CM have rarely been reported. In this study, the findings revealed that PSB4 (injection) could effectively protect clinical mastitis in dairy cows. Specifically, the milk SCC was reduced. Furthermore, the cure rate can reach more than 70%, the effective rate is higher than 90%, and the recurrence rate is very low. Additionally, the body temperature of the mastitis cows decreased and returned to normal under PSB4 treatment; the decreased milk yield of also rebounded significantly and returned to the production level of healthy cows. The results showed that PSB4 had good comprehensive efficacy, and compared with the antibiotic treatment group, the CM cows treated with PSB4 had a lower recurrence rate, was an ideal drug for treating cow mastitis.

*Staphylococcus aureus*, *Streptococcus* and *Escherichia coli* are the main pathogens causing mastitis in dairy cows [42,43], which can invade breast tissue and release toxic substances, such as lipopolysaccharides and exotoxin [44–46]. The pathogenic microorganisms were detected in milk of diseased cows on the farm by using a 16-linked bovine mastitis pathogen nucleic acid detection kit. Previous studies have been shown that PSB4 can inhibit pathogenic microorganisms such as *Staphylococcus aureus* and *Colibacillus* [47–49]. In the current study, *Streptococcus agalactiae*, *Staphylococcus aureus*, *Enterococcus*, *Colibacillus*, and *yeast* all had a high negative conversion rate, indicating that PSB4 had a good inhibitory effect on CM pathogens. This shows a similar effect with other saponins [50,51].

Cytokines play an important role in immune defense and inflammation. When pathogenic microorganisms invade breast tissue, immune cells will produce and release various inflammatory factors, causes epithelial cell damage. Once the cell barrier is destroyed, it leads to inflammation and shows obvious CM signs. Research has shown that Pulsatilla decoction and its main components can inhibit the high-level secretion of IL-1β, IL-6, and endothelin-1 (ET-1) in endothelial cells induced by endotoxin, which has potential antibacterial effect [50]. Additionally, studies have shown that many plant saponins have the effect of reducing inflammatory factors and alleviating inflammation [52,53], and PSB4 shows a similar effect, it has been reported that it can downregulate the release of IL-1β, IL-6, and TNF-α in mice with ulcerative colitis and has anti-inflammatory and immunoregulatory effects [33]. In this study, the levels of TNF-α, IL-1α, IL-1β, IL-6, and IL-8 in the serum of dairy cows with mastitis were higher than those in healthy cows, however, after 7 days of treatment, these indicators basically returned to the level of the healthy group, which is consistent with the results of previous studies [54,55]. These findings suggest that PSB4 can reduce the inflammatory response of diseased cows by inhibiting the expression of inflammatory factors, thereby alleviating the development of breast inflammation.

To study the effect of PSB4 on immune factors in the serum of cows with clinical mastitis, the levels of IgA, IgG, and IgM were measured. IgA, IgG, and IgM, the main components of immunoglobulins, play an important role in enhancing immunity, assisting immune cells in engulfing pathogenic microorganisms, and neutralizing pathogenic microbial toxins [56,57]. Furthermore, the joint action of various immune factors could resist the invasion of pathogenic microorganisms, regulate inflammatory responses, and maintain immune defense function. Previous studies have shown that Pulsatilla and Pulsatilla saponins can increase the levels of IgG and IgM and improve the immune function and antioxidant properties in weaned piglets and mice [58]. However, the regulation of IgA, IgG and IgM on the body is two-way. When these indicators are low, the body is in a state of low immunity, and when these indicators continue to be high, the body may be in a serious infection or related to autoimmune diseases. Therefore, when the levels of IgA, IgG and IgM fluctuate within the normal range, it is the body’s normal immune response to bacteria, viruses, etc., but the abnormal fluctuations in values are non-healthy. In this study, the levels of IgA, IgG and IgM in the serum of CM cows were higher than those in the healthy group before treatment. This may be due to the excessive inflammation of mastitis cows. Conversely, after treatment with experimental drugs (PSB4), it decreased significantly and approached the level of healthy cows. These results indicate that PSB4 can effectively alleviate the immune storm of CM cows and inhibit the development of breast inflammation.

Reactive oxygen species (ROS) have a dual role in the body, an appropriate level of ROS can enhance immunity, eliminate bacteria, and kill malignant cells, however, excessive content will damage normal cells and aggravate the development of the disease. In the process of inflammation, mast cells and white blood cells are recruited to the damaged site, due to the increase of oxygen uptake, the release and accumulation of ROS are increased in the damaged site, resulting in “respiratory burst”. Excessive ROS can destroy cellular proteins, lipids and DNA, leading to cell damage. These damaged cells are removed by the body, causing the production of endogenous damage associated molecular patterns and the release of cytokines. The release of cytokines increases, recruiting and activating more inflammatory cells, causing systemic inflammatory response. Therefore, when the antioxidant capacity is not enough to remove ROS, the mammary gland tissue is damaged to develop mastitis [59–62]. T-AOC, SOD, MDA, and GSH-Px are the main indicators of antioxidant function. T-AOC is a comprehensive index to measure the antioxidant capacity, which can judge the overall degree of oxidative stress. SOD can alleviate the oxidative damage by scavenging strong oxidizing substances such as O_2_^−^ and hydrogen peroxide. MDA is one of the important indicators of the degree of damage to lipids attacked by oxygen free radicals. GSH-Px is an important lipid peroxide decomposition enzyme, it is an important index to measure the antioxidant capacity [63,64]. A large number of previous studies have shown that a variety of plant saponins show good antioxidant and organ protection effects [65–68]. In this study we observed that the levels of T-AOC, SOD, and GSH-Px in the serum of CM cows before treatment were lower than those of NC group cows, whereas the MDA level was higher than those of NC group, which is consistent with previous studies [58]. This may,be because mastitis cows produce excessive O_2_^−^ and H_2_O_2_ to fight the inflammatory response; however, excessive oxygen free radicals lead to lipid peroxidation damage and decreased levels of antioxidant enzymes. Conversely, the T-AOC, SOD, and GSH-Px levels in the serum of CM cows were increased, while the MDA level decreased significantly after PSB4 treatment. In addition, the results of this experiment showed that after treatment, the levels of T-AOC and SOD in PSB4 group were higher than those in PC group. In view of these results, PSB4 could enhance the antioxidant capacity of CM cows, and was superior to antibiotics in improving antioxidant capacity, making it an ideal drug for the treatment of CM cows.

White blood cells are important immune cells, and their main role is to devour bacteria for disease prevention [69]. The CM cows selected in this experiment were in an inflammatory condition, and the WBC count was significantly higher than the normal value. While PSB4 and ceftiofur sodium inhibited pathogenic bacteria to alleviate inflammation after treatment. Regarding biochemical parameters, there were no marked differences in the levels of ALP, ALT, AST, UREA, CREA, TP, LDH, and CK before and after treatment between the NC group and the drug-treated group. However, the ALB level of CM cows was low, increased and returned to normal after treatment, while the effects of PSB4 and ceftiofur sodium on ALB level were similar. These results indicate that PSB4 can improve the anti-inflammatory ability of CM dairy cows without toxic and side effects.

## Conclusions

The results of this study show that PSB4 has noticeable effect on clinical mastitis in dairy cows. PSB4 can effectively restore milk production, reduce SCC in milk, inhibit mastitis pathogens, alleviate the inflammatory response, enhance immunity, improve the antioxidant capacity, and restore some blood routine and blood biochemical indicators. In summary, PSB4 has the characteristics of anti-inflammatory, anti-oxidation, low toxicity and not easy to produce drug resistance. It has great potential and research value to replace antibiotics in the treatment of mastitis.

## Supporting information

S1 File Raw data. (XLSX)

## References

[1] Rifa’i RLE, Hawa LC, Surjowardojo P. Prevalence of subclinical mastitis in Holstein-Friesian cow dairy among small-scale farms in Batu, Indonesia. International Journal of Veterinary Science. 2024;13(5):592–5. doi: 10.47278/journal.ijvs/2024.144

[2] Oliveira L, Hulland C, Ruegg PL. Characterization of clinical mastitis occurring in cows on 50 large dairy herds in Wisconsin. Journal of Dairy Science. 2013;96(12):7538–49. doi: 10.3168/jds.2012-6078 24119795

[3] Dittrich I, Gertz M, Krieter J. Alterations in sick dairy cows’ daily behavioural patterns. Heliyon. 2019;5(11):e02902. doi: 10.1016/j.heliyon.2019.e02902 31799469 PMC6881618

[4] Kayano M, Itoh M, Kusaba N, Hayashiguchi O, Kida K, Tanaka Y, et al. Associations of the first occurrence of pathogen-specific clinical mastitis with milk yield and milk composition in dairy cows. J Dairy Res. 2018;85(3):309–16. doi: 10.1017/S0022029918000456 30101726

[5] Brandt M, Haeussermann A, Hartung E. Invited review: technical solutions for analysis of milk constituents and abnormal milk. J Dairy Sci. 2010;93(2):427–36. doi: 10.3168/jds.2009-2565 20105515

[6] Gomes F, Henriques M. Control of Bovine Mastitis: Old and Recent Therapeutic Approaches. Curr Microbiol. 2016;72(4):377–82. doi: 10.1007/s00284-015-0958-8 26687332

[7] Ruegg PL. A 100-Year Review: Mastitis detection, management, and prevention. J Dairy Sci. 2017;100(12):10381–97. doi: 10.3168/jds.2017-13023 29153171

[8] Avanus K, Yilmaz A, Güneş H, Altinel A, Ekiz B, Yalçintan H, et al. CXCR1 Gene SNP Variability that Affects Mastitis Resistance in Holstein Cows in Türkiye. Kafkas Univ Vet Fak Derg. 2024. doi: 10.9775/kvfd.2024.32948

[9] Kamal M, Martinez-Boggio G, Rafiq N, Yu Y, Peñagaricano F, Usman T. Genetic Association of Candidate Genes with Milk and Mastitis Resistance Traits using SNP-Chip Array in Holstein Friesian and Pakistani Indigenous Dairy Cattle Breeds. PAK VET J. 2025;45(1):402–8. doi: 10.29261/pakvetj/2025.119

[10] Ghazvineh N, Mokhtari A, Ghorbanpoor Najaf Abadi M, Kadivar A, Shrokh Shahki S. Molecular Detection of Selective Virulence Factors of Mycoplasma bovis Local Isolates Involved in Bovine Mastitis. Kafkas Univ Vet Fak Derg. 2024. doi: 10.9775/kvfd.2024.32118

[11] Abdalhamed AM, Zeedan GSG, Hafez AAN. Rapid on-site detection of major mastitis pathogens in ruminants using a colorimetric loop-mediated isothermal amplification assay. International Journal of Veterinary Science. 2024;13(2):186–94. doi: 10.47278/journal.ijvs/2023.081

[12] Klaas IC, Zadoks RN. An update on environmental mastitis: Challenging perceptions. Transbound Emerg Dis. 2018;65 Suppl 1:166–85. doi: 10.1111/tbed.12704 29083115

[13] Wellnitz O, Bruckmaier RM. The innate immune response of the bovine mammary gland to bacterial infection. Vet J. 2012;192(2):148–52. doi: 10.1016/j.tvjl.2011.09.013 22498784

[14] Ruegg PL. Making Antibiotic Treatment Decisions for Clinical Mastitis. Vet Clin North Am Food Anim Pract. 2018;34(3):413–25. doi: 10.1016/j.cvfa.2018.06.002 30316500

[15] Stevens M, Piepers S, De Vliegher S. Mastitis prevention and control practices and mastitis treatment strategies associated with the consumption of (critically important) antimicrobials on dairy herds in Flanders, Belgium. Journal of Dairy Science. 2016;99(4):2896–903. doi: 10.3168/jds.2015-10496 26874421

[16] Tenhagen B-A, Köster G, Wallmann J, Heuwieser W. Prevalence of mastitis pathogens and their resistance against antimicrobial agents in dairy cows in Brandenburg, Germany. J Dairy Sci. 2006;89(7):2542–51. doi: 10.3168/jds.S0022-0302(06)72330-X 16772573

[17] Krömker V, Leimbach S. Mastitis treatment-Reduction in antibiotic usage in dairy cows. Reprod Domest Anim. 2017;52 Suppl 3:21–9. doi: 10.1111/rda.13032 28815847

[18] Maity S, Ambatipudi K. Mammary microbial dysbiosis leads to the zoonosis of bovine mastitis: a One-Health perspective. FEMS Microbiology Ecology. 2020;97(1). doi: 10.1093/femsec/fiaa241 33242081

[19] Goulart DB, Mellata M. Escherichia coli Mastitis in Dairy Cattle: Etiology, Diagnosis, and Treatment Challenges. Front Microbiol. 2022;13:928346. doi: 10.3389/fmicb.2022.928346 35875575 PMC9301288

[20] Celebi D, Celebi O, Baser S, Ari B, Ektas Kalayci S, Kara A. Efficacy of Pyocyanin Isolated from Pseudomonas aeruginosa and Lactobacillus plantarum Against Methicillin Resistant Staphylococcus aureus Caused Bovine Mastitis. Kafkas Univ Vet Fak Derg. 2025. doi: 10.9775/kvfd.2024.33429

[21] Sindhu S, Saini T, Rawat HK, Chahar M, Grover A, Ahmad S, et al. Beyond conventional antibiotics approaches: Global perspectives on alternative therapeutics including herbal prevention, and proactive management strategies in bovine mastitis. Microb Pathog. 2024;196:106989. doi: 10.1016/j.micpath.2024.106989 39357684

[22] Lago A, Godden SM. Use of Rapid Culture Systems to Guide Clinical Mastitis Treatment Decisions. Vet Clin North Am Food Anim Pract. 2018;34(3):389–412. doi: 10.1016/j.cvfa.2018.06.001 30316499

[23] Yang H, Chen X, Jiang C, He K, Hu Y. Antiviral and Immunoregulatory Role Against PCV2 in Vivo of Chinese Herbal Medicinal Ingredients. J Vet Res. 2017;61(4):405–10. doi: 10.1515/jvetres-2017-0062 29978102 PMC5937337

[24] Zheng S, Zheng H, Zhang R, Piao X, Hu J, Zhu Y, et al. Immunomodulatory Effect of Ginsenoside Rb2 Against Cyclophosphamide-Induced Immunosuppression in Mice. Front Pharmacol. 2022;13:927087. doi: 10.3389/fphar.2022.927087 35814238 PMC9263391

[25] Liu J, Wu Y, Ma W, Zhang H, Meng X, Zhang H, et al. Anti-Inflammatory Activity of Panax notoginseng Flower Saponins Quantified Using LC/MS/MS. Molecules. 2023;28(5):2416. doi: 10.3390/molecules28052416 36903661 PMC10005202

[26] Sonfack G, Fossi Tchinda C, Simo IK, Bitchagno GTM, Nganou BK, Çelik İ, et al. Saponin with antibacterial activity from the roots of Albizia adianthifolia. Nat Prod Res. 2021;35(17):2831–9. doi: 10.1080/14786419.2019.1672689 31583912

[27] Wang W, Kong M, Shen F, Li P, Chen C, Li Y, et al. Ginsenoside Rg3 targets glycosylation of PD-L1 to enhance anti-tumor immunity in non-small cell lung cancer. Front Immunol. 2024;15:1434078. doi: 10.3389/fimmu.2024.1434078 39247194 PMC11377313

[28] Deng Y, Zhang Y, Liu G, Zhou P, Li P, Zhao Z, et al. Saponins from Momordica charantia exert hypoglycemic effect in diabetic mice by multiple pathways. Food Science & Nutrition. 2023;11(12):7626–37. doi: 10.1002/fsn3.3682 38107145 PMC10724611

[29] He L, Zhang Y, Kang N, Wang Y, Zhang Z, Zha Z, et al. Anemoside B4 attenuates nephrotoxicity of cisplatin without reducing anti-tumor activity of cisplatin. Phytomedicine. 2019;56:136–46. doi: 10.1016/j.phymed.2018.10.035 30668334

[30] Kang N, Shen W, Zhang Y, Su Z, Yang S, Liu Y, et al. Anti-inflammatory and immune-modulatory properties of anemoside B4 isolated from Pulsatilla chinensis in vivo. Phytomedicine. 2019;64:152934. doi: 10.1016/j.phymed.2019.152934 31454651

[31] Li S, Li X, Yang R, Wang B, Li J, Cao L, et al. Effects of anemoside B4 on pharmacokinetics of florfenicol and mRNA expression of CXR, MDR1, CYP3A37 and UGT1E in broilers. J Vet Med Sci. 2019;81(12):1804–9. doi: 10.1292/jvms.19-0293 31611492 PMC6943327

[32] Wang S, Tang S, Chen X, Li X, Jiang S, Li H-P, et al. Pulchinenoside B4 exerts the protective effects against cisplatin-induced nephrotoxicity through NF-κB and MAPK mediated apoptosis signaling pathways in mice. Chem Biol Interact. 2020;331:109233. doi: 10.1016/j.cbi.2020.109233 32991863

[33] Ma H, Zhou M, Duan W, Chen L, Wang L, Liu P. Anemoside B4 prevents acute ulcerative colitis through inhibiting of TLR4/NF-κB/MAPK signaling pathway. Int Immunopharmacol. 2020;87:106794. doi: 10.1016/j.intimp.2020.106794 32688280

[34] Liang L, Cao W, Li L, Liu W, Wei X, Chen J, et al. Fabrication and characterisation of pea protein isolate‐chlorogenic acid nanoparticles. Int J of Food Sci Tech. 2024;59(3):1615–23. doi: 10.1111/ijfs.16911

[35] Zhang Y, Zha Z, Shen W, Li D, Kang N, Chen Z, et al. Anemoside B4 ameliorates TNBS-induced colitis through S100A9/MAPK/NF-κB signaling pathway. Chin Med. 2021;16(1):11. doi: 10.1186/s13020-020-00410-1 33461587 PMC7814617

[36] Kaseke TB, Chikwambi Z, Gomo C, Mashingaidze AB, Murungweni C. Antibacterial activity of medicinal plants on the management of mastitis in dairy cows: A systematic review. Vet Med Sci. 2023;9(6):2800–19. doi: 10.1002/vms3.1268 37725398 PMC10650345

[37] Arbab S, Ullah H, Bano I, Li K, Ul Hassan I, Wang W, et al. Evaluation of in vitro antibacterial effect of essential oil and some herbal plant extract used against mastitis pathogens. Vet Med Sci. 2022;8(6):2655–61. doi: 10.1002/vms3.959 36253877 PMC9677380

[38] Burmańczuk A, Hola P, Milczak A, Piech T, Kowalski C, Wojciechowska B, et al. Quercetin decrease somatic cells count in mastitis of dairy cows. Res Vet Sci. 2018;117:255–9. doi: 10.1016/j.rvsc.2018.01.006 29331686

[39] Guo-Wang L. Bacteriostatic effect of madder and etc. on pathogenic bacteria in dairy cows with mastitis. J CoSL. 2010.

[40] Hu G, Wang J, Hong D, Zhang T, Duan H, Mu X, et al. Effects of aqueous extracts of Taraxacum Officinale on expression of tumor necrosis factor-alpha and intracellular adhesion molecule 1 in LPS-stimulated RMMVECs. BMC Complement Altern Med. 2017;17(1):38. doi: 10.1186/s12906-016-1520-3 28077102 PMC5225575

[41] Zhang D, Jin G, Liu W, Dou M, Wang X, Shi W, et al. Salvia miltiorrhiza polysaccharides ameliorates Staphylococcus aureus-induced mastitis in rats by inhibiting activation of the NF-κB and MAPK signaling pathways. BMC Vet Res. 2022;18(1):201. doi: 10.1186/s12917-022-03312-6 35624447 PMC9137159

[42] Biosecurity Measures, Bacterial Prevalence and Economic Implications of Environmental Mastitis and Hygienic Milking Practices on an Egyptian Dairy Farm. Int J Vet Sci. 2024. doi: 10.47278/journal.ijvs/2024.193

[43] Metagenomic Analysis of Bacterial Diversity in Milk of Mastitis Cows from Farms with Different Milking Management. Int J Vet Sci. 2024. doi: 10.47278/journal.ijvs/2024.245

[44] Klibi A, Jouini A, Boubaker El Andolsi R, Kmiha S, Ben Hamda C, Ghedira K, et al. Epidemiology of β-Lactamase-Producing Staphylococci and Gram Negative Bacteria as Cause of Clinical Bovine Mastitis in Tunisia. Biomed Res Int. 2019;2019:2165316. doi: 10.1155/2019/2165316 31534954 PMC6732581

[45] Ali T, Kamran RA, Wazir I, Ullah R, Shah P, et al. Prevalence of Mastitis Pathogens and Antimicrobial Susceptibility of Isolates From Cattle and Buffaloes in Northwest of Pakistan. Front Vet Sci. 2021;8:746755. doi: 10.3389/fvets.2021.746755 34722707 PMC8551922

[46] Tora ET, Bekele NB, Suresh Kumar RS. Bacterial profile of bovine mastitis in Ethiopia: a systematic review and meta-analysis. PeerJ. 2022;10:e13253. doi: 10.7717/peerj.13253 35547189 PMC9083533

[47] Hu Y, Mao A, Tan Y, Zhao Y, He K. Role of 5 Saponins in Secretion of Cytokines by PRRSV-induced Endothelial Cells. Drug Res (Stuttg). 2016;66(7):357–62. doi: 10.1055/s-0042-106577 27144658

[48] Yuan R, He J, Huang L, Du L-J, Gao H, Xu Q, et al. Anemoside B4 Protects against Acute Lung Injury by Attenuating Inflammation through Blocking NLRP3 Inflammasome Activation and TLR4 Dimerization. J Immunol Res. 2020;2020:7502301. doi: 10.1155/2020/7502301 33344657 PMC7732379

[49] Zhong J, Tan L, Chen M, He C. Pharmacological activities and molecular mechanisms of Pulsatilla saponins. Chin Med. 2022;17(1):59. doi: 10.1186/s13020-022-00613-8 35606807 PMC9125917

[50] Hu Y, Chen X, Duan H, Hu Y, Mu X. Pulsatilla decoction and its active ingredients inhibit secretion of NO, ET-1, TNF-alpha, and IL-1 alpha in LPS-induced rat intestinal microvascular endothelial cells. Cell Biochem Funct. 2009;27(5):284–8. doi: 10.1002/cbf.1570 19472295

[51] Thomas FC, Geraghty T, Simões PBA, Mshelbwala FM, Haining H, Eckersall PD. A pilot study of acute phase proteins as indicators of bovine mastitis caused by different pathogens. Res Vet Sci. 2018;119:176–81. doi: 10.1016/j.rvsc.2018.06.015 29945037

[52] Zheng Y, Tan H, Chai J, Han L, Zhai C, Lee J, et al. Ginseng fruit rare saponins (GFRS) improved inflammatory response: In vitro and in vivo assessment. Fitoterapia. 2024;179:106244. doi: 10.1016/j.fitote.2024.106244 39396651

[53] Xue L, Hu M, Zhu Q, Li Y, Zhou G, Zhang X, et al. GRg1 inhibits the TLR4/NF-kB signaling pathway by upregulating miR-216a-5p to reduce growth factors and inflammatory cytokines in DR. Mol Biol Rep. 2023;50(11):9379–94. doi: 10.1007/s11033-023-08895-3 37819496 PMC10635910

[54] Piotrowska-Tomala KK, Bah MM, Jankowska K, Lukasik K, Warmowski P, Galvao AM, et al. Lipopolysaccharides, cytokines, and nitric oxide affect secretion of prostaglandins and leukotrienes by bovine mammary gland during experimentally induced mastitis in vivo and in vitro. Domest Anim Endocrinol. 2015;52:90–9. doi: 10.1016/j.domaniend.2015.03.001 25935895

[55] Shah KN, Valand P, Nauriyal DS, Joshi CG. Immunomodulation of IL-1, IL-6 and IL-8 cytokines by Prosopis juliflora alkaloids during bovine sub-clinical mastitis. 3 Biotech. 2018;8(10):409. doi: 10.1007/s13205-018-1438-1 30237956 PMC6138006

[56] Xuan X, Zhao P, Zhang C, Shi Y. Progress on defense mechanism of lacteal gland in cow. Journal of Agricultural Sciences. 2005;26(2):81–6.

[57] Karagiannis SN, Karagiannis P, Josephs DH, Saul L, Gilbert AE, Upton N, et al. Immunoglobulin E and Allergy: Antibodies in Immune Inflammation and Treatment. Microbiol Spectr. 2013;1(1):10.1128/microbiolspec.AID-0006–2012. doi: 10.1128/microbiolspec.AID-0006-2012 26184813

[58] Jin C. Effects of Pulsatilla chinensis and its active ingredient-saponin on immunity and antioxidation of weaned piglets. Pratacultural Science. 2015.

[59] Limón-Pacheco J, Gonsebatt ME. The role of antioxidants and antioxidant-related enzymes in protective responses to environmentally induced oxidative stress. Mutat Res. 2009;674(1–2):137–47. doi: 10.1016/j.mrgentox.2008.09.015 18955158

[60] Dunnill C, Patton T, Brennan J, Barrett J, Dryden M, Cooke J, et al. Reactive oxygen species (ROS) and wound healing: the functional role of ROS and emerging ROS-modulating technologies for augmentation of the healing process. Int Wound J. 2017;14(1):89–96. doi: 10.1111/iwj.12557 26688157 PMC7950185

[61] Pinegin B, Vorobjeva N, Pashenkov M, Chernyak B. The role of mitochondrial ROS in antibacterial immunity. J Cell Physiol. 2018;233(5):3745–54. doi: 10.1002/jcp.26117 28771715

[62] Gwozdzinski K, Pieniazek A, Gwozdzinski L. Reactive Oxygen Species and Their Involvement in Red Blood Cell Damage in Chronic Kidney Disease. Oxid Med Cell Longev. 2021;2021:6639199. doi: 10.1155/2021/6639199 33708334 PMC7932781

[63] Paździor-Czapula K, Gesek M, Rotkiewicz T, Kluciński W, Kołodziejska J, Kleczkowski M, et al. Immunohistochemical evaluation of superoxide dismutase (Cu/Zn SOD) concentrations in erythrocytes of dairy cattle and farm-raised deer by a computer-assisted analysis of microscopic images. Pol J Vet Sci. 2014;17(2):275–9. doi: 10.2478/pjvs-2014-0038 24988853

[64] Mavangira V, Sordillo LM. Role of lipid mediators in the regulation of oxidative stress and inflammatory responses in dairy cattle. Res Vet Sci. 2018;116:4–14. doi: 10.1016/j.rvsc.2017.08.002 28807478

[65] Li H, Zhai B, Sun J, Fan Y, Zou J, Cheng J, et al. Antioxidant, Anti-Aging and Organ Protective Effects of Total Saponins from Aralia taibaiensis. Drug Des Devel Ther. 2021;15:4025–42. doi: 10.2147/DDDT.S330222 34594101 PMC8476322

[66] Li J, Sun Y, Yang N, Zhang H, Hu Y, Wang H, et al. Protective effects of maternal administration of total saponins of Codonopsis pilosula in the mice offspring following diarrhea: role of immune function, antioxidant function, and intestinal inflammatory injury. Environ Sci Pollut Res Int. 2023;30(53):113903–16. doi: 10.1007/s11356-023-30281-6 37858017

[67] Liu S, Zhu X, Pei H, Zhao Y, Zong Y, Chen W, et al. Ginseng Stem-and-Leaf Saponins Mitigate Chlorpyrifos-Evoked Intestinal Toxicity In Vivo and In Vitro: Oxidative Stress, Inflammatory Response and Apoptosis. Int J Mol Sci. 2023;24(21):15968. doi: 10.3390/ijms242115968 37958950 PMC10650881

[68] Li J, Han J, Lv J, Wang S, Qu L, Jiang Y. Saikosaponin A-Induced Gut Microbiota Changes Attenuate Severe Acute Pancreatitis through the Activation of Keap1/Nrf2-ARE Antioxidant Signaling. Oxid Med Cell Longev. 2020;2020:9217219. doi: 10.1155/2020/9217219 33204401 PMC7652616

[69] Wang R-H, Wen W-X, Jiang Z-P, Du Z-P, Ma Z-H, Lu A-L, et al. The clinical value of neutrophil-to-lymphocyte ratio (NLR), systemic immune-inflammation index (SII), platelet-to-lymphocyte ratio (PLR) and systemic inflammation response index (SIRI) for predicting the occurrence and severity of pneumonia in patients with intracerebral hemorrhage. Front Immunol. 2023;14:1115031. doi: 10.3389/fimmu.2023.1115031 36860868 PMC9969881

